# Is it safer to perform surgery before or after liver transplantation? A case-match study for colorectal and small-bowel surgery

**DOI:** 10.1007/s00423-025-03858-7

**Published:** 2025-09-27

**Authors:** Phil Meister, Roxana Pantea, Samira Vestweber, Marc A Reschke, Ulf Neumann, Andreas D Rink

**Affiliations:** Department of General-, Visceral-, Vascular-and Transplantation Surgery, University Hospital Essen, Essen, Germany

**Keywords:** Cirrhosis, Surgery, Transplantation, Perioperative risk

## Abstract

**Aims:**

Surgical risks are elevated in both patients with cirrhosis and in liver transplant recipients. We aimed to quantify surgical outcomes in comparable patients and procedures.

**Methods:**

This case-control study included liver transplant recipients and cirrhosis patients who underwent small bowel or colorectal surgery. Patients were matched based on Charlson Comorbidity Index (CCI) (± 1), age (± 5 years), and surgical modality. In-hospital mortality, length of hospital stay (LOS), and major morbidity (Dindo-Clavien grade ≥ 3) were used as outcome criteria.

**Results:**

45 cirrhosis and 45 matched transplant patients were included. Mean age and CCI were 65 years and 6.3, respectively. 38% of all patients underwent emergency surgery. Mortality was significantly higher in the cirrhosis group (38% vs. 11%, *p* = 0.003). Stratification of cirrhosis patients by MELD revealed no significant difference between patients with MELD ≤ 14 and transplant recipients. However, patients with MELD > 14 exhibited substantially increased mortality (64% vs. 9%, *p* = 0.07, ns).

**Conclusions:**

Colorectal and small bowel surgery in both cirrhosis and transplant patients carries significant risks. Mortality was significantly higher in cirrhosis patients overall, but data suggests the risk of surgery in cirrhosis patients with MELD scores ≤ 14 might be comparable to transplant patients, while those with MELD scores > 14 are at particular risk. Small sample size and heterogeneity of procedures limit these findings; still, the necessity of surgery in patients with higher MELD should be carefully evaluated, as delaying surgery until after liver transplantation may be safer.

***What does this paper add to literature?*:**

This paper contains the largest case-matched comparison of surgery in cirrhosis patients with liver transplant patients. We quantify the risk for small bowel and colorectal surgery in comparable patients for the first time, to assist clinical decision of potentially delaying surgery until after liver transplantation.

**Supplementary Information:**

The online version contains supplementary material available at 10.1007/s00423-025-03858-7.

## Introduction

Liver cirrhosis, a severe condition, often necessitates liver transplantation for definitive treatment. Surgical interventions in cirrhotic patients can precipitate acute decompensation or acute-on-chronic liver failure, life-threatening events characterized by multi-organ failure [1]. The inherent risk associated with surgery is significantly elevated in cirrhotic patients, even for minor procedures [2, 3]. The Model for End-Stage Liver Disease (MELD) score, initially developed to predict mortality in cirrhotic patients [4]has proved useful for perioperative risk assessment in this population [5]. A MELD score exceeding 20 is associated with a 50% perioperative mortality rate [6]and a linear correlation between MELD score and perioperative morbidity has been observed [7], with a 20% increase in in-hospital mortality per unit increase in MELD score [3]. Given these elevated risks, surgical interventions in cirrhotic patients are ideally avoided or delayed until after liver transplantation. However, surgery in liver transplant recipients also presents unique challenges and risks, and comprehensive data on the actual surgical risk in this specific population are limited. Retrospective studies have demonstrated a relevantly increased surgical risk in kidney transplant recipients [8, 9] and an elevated risk of colectomy due to diverticulitis in immunocompromised patients [10–12].

The indication for surgery in cirrhotic patients, particularly those awaiting liver transplantation, therefore remains a significant clinical challenge. This study aimed to quantify surgical outcomes in comparable patients and procedures to inform clinical decision-making and optimize patient management.

## Methods

A retrospective analysis was conducted using data extracted from the digital hospital information system for all patients diagnosed with either cirrhosis or having undergone previous liver transplantation who underwent small bowel or colorectal surgery at the University Hospital Essen, Germany, between January 2008 and October 2024. The study was approved by the local ethics committee (8-8348-BO & 24-12246-BO) and adhered to the Declaration of Helsinki.

Patient characteristics, including sex, age, relevant laboratory values (e.g. bilirubin, INR and creatinine to calculate MELD score), and comorbidities, were recorded. Surgical procedures were categorized as elective or emergency. Outcomes were assessed by in-hospital mortality, length of hospital stay (LOS), and major morbidity (Dindo-Clavien ≥ 3a). A case-matched analysis was performed, matching cirrhotic patients with transplant recipients based on surgical modality, Charlson Comorbidity Index (CCI ± 1), and age (± 5 years). Cirrhosis patients and their matched counterparts were further stratified by the cirrhotic patients’ MELD score (low < 10, mid 10–14, high > 14), in accordance with previous findings [3]. Only patients with complete dataset were included; patients with multiorgan transplant or recurrent cirrhosis after transplantation were excluded.

Statistical analyses were performed using SPSS 29.0 software (IBM Inc., Armonk, NY, USA). Continuous variables were tested for normal distribution via Kolmogorov-Smirnov test and then appropriately compared using a two-sided t-test or Mann-Whitney U-test (length of hospital stay). 95% confidence intervals (CI) were calculated. Data are presented as mean ± standard deviation or median [range], as appropriate. A p-value < 0.05 was considered statistically significant.

## Results

A total of 45 cirrhotic patients were matched with 45 liver transplant recipients. Both groups had a mean age of 62.5 years and a mean Charlson Comorbidity Index (CCI) of 6.3. Emergency surgery was performed in 17 patients (38%) in each group. The cirrhotic group comprised 28 males (62%), while the transplant group had 23 males (51%). The mean MELD score in the cirrhotic group was 13.7 ± 8.5, and the mean time from transplant to surgery for the transplant group was 8.5 ± 8.0 years.

Indications for surgery differed between the groups. In cirrhotic patients, the most common indications were malignancy (42%), perforation (29%), obstruction (11%), and inflammation (11%). For transplant patients, the leading causes were obstruction (26%), malignancy (20%), perforation (18%), and inflammation (18%).

While surgical procedures were heterogeneous and not part of the matching criteria, the most common procedures across both groups were small bowel resection (38%), right-sided hemicolectomy (20%), sigmoid resection (16%), and total colectomy (10%). Detailed patient characteristics, indications, and procedures are presented in Table 1.

**Table 1:** Patients’ characteristics, indications and procedures

	Overall *n* = 90	Cirrhosis *n* = 45	Transplantation *n* = 45	
Sex (male)	51 (57%)	28 (62%)	23 (51%)	
Age (mean ± SD)	62.5 ± 13.2	62.5 ± 12.6	62.5 ± 13.8	
CCI (mean ± SD)	6.2 ± 2.1	6.3 ± 2.2	6.1 ± 2.0	
Malignancy	28 (31%)	19 (42%)	9 (20%)	
Perforation	21 (23%)	13 (29%)	8 (18%)	
Obstruction	17 (19%)	5 (11%)	12 (26%)	
Bleeding	3 (3%)	1 (2%)	2 (4%)	
Inflammation	13 (14%)	5 (11%)	8 (18%)	
Other	8 (9%)	2 (4%)	6 (13%)	
Small bowel resection	34 (38%)	13 (29%)	21 (46%)	
Hemicolectomy right	18 (20%)	13 (29%)	5 (11%)	
Sigma resection	14 (16%)	9 (20%)	5 (11%)	
Total colectomy	9 (10%)	3 (7%)	6 (13%)	
Other	15 (17%)	7 (16%)	8 (18%)	

In-hospital mortality was significantly higher in the cirrhotic group (38% vs. 11%, *p* = 0.003). Additionally, the length of hospital stay (LOS) was significantly longer for cirrhotic patients (20.6 vs. 15.7 days, *p* = 0.01). There was no significant difference in major morbidity (Dindo-Clavien ≥ 3a) between the groups (53% vs. 42%, *p* = 0.29, ns).

For elective surgery (*n* = 28 patients per group), cirrhotic patients showed a non-statistically significant trend towards worse outcomes: mortality (25% vs. 14%, *p* = 0.3, ns), major morbidity (43% vs. 32%, *p* = 0.4, ns), and LOS (17.9 vs. 13.3 days, *p* = 0.08, ns).

Emergency surgery (*n* = 17 patients per group) resulted in significantly worse outcomes for cirrhotic patients: mortality (59% vs. 6%, *p* = 0.001), major morbidity (71% vs. 59%, *p* = 0.48, ns), and LOS (28.3 vs. 19.3 days, *p* = 0.047).

**Fig. 1:**
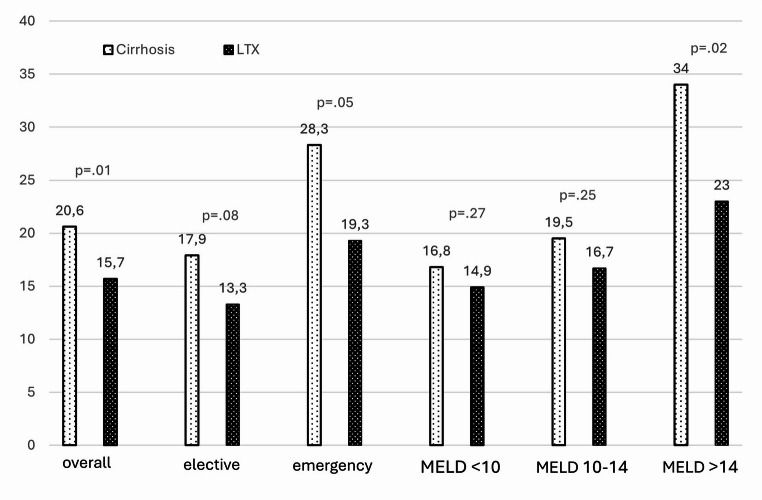
Length of hospital stay in days

**Fig. 2:**
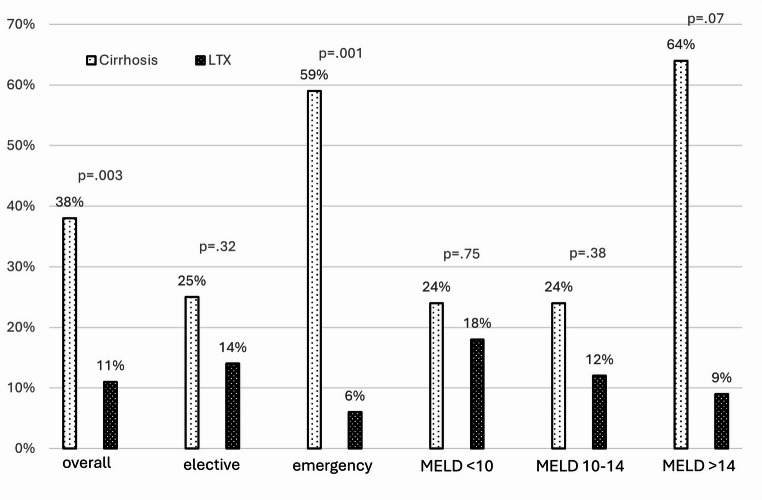
in-hospital mortality in percentage

Cirrhotic patients were further stratified by MELD score. Patients with MELD scores < 10 (*n* = 17) had comparable outcomes to transplant recipients (mortality: 24% vs. 18% *p* = 0.75; morbidity: 35% vs. 41% *p* = 0.63; LOS: 16.8 vs. 14.9 days *p* = 0.27). Patients with MELD scores 10–14 (*n* = 17) also showed similar outcomes to transplant recipients (mortality: 24% vs. 12% *p* = 0.3; morbidity: 41% vs. 41% *p* = 1; LOS: 19.5 vs. 16.7 days *p* = 0.25). However, patients with MELD scores > 14 (*n* = 11) had significantly worse outcomes (mortality: 64% vs. 9%, *p* = 0.07 ns; morbidity: 82% vs. 55%, *p* = 0.018; LOS: 34 vs. 23 days *p* = 0.02 (See Figure 1, 2, & 3) and (Table 2).

**Fig. 3:**
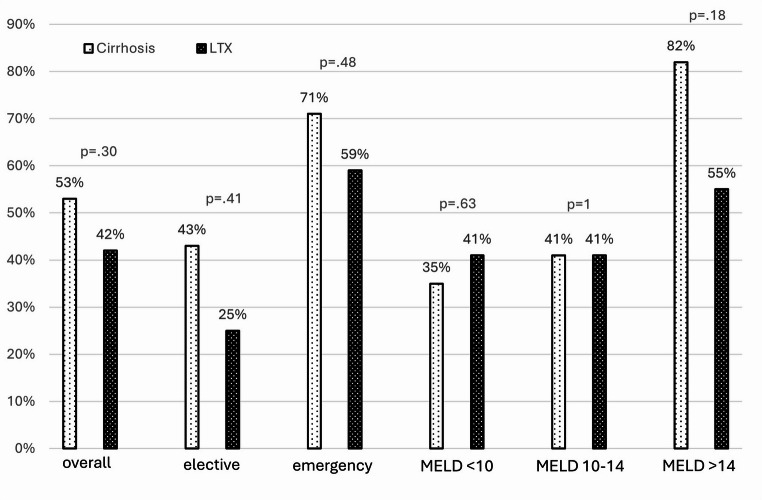
morbidity (Dindo Clavien ≥ 3) in percentage

**Table 2:** Patient outcomes

	Overall n=90	Cirrhosis n=45	Transplantation n=45	p
Length of hospital stay (days, mean±SD)	17.7 ± 14.2	20.6±11.7	15.7±15.8	0.010
Morbidity (n, %)	43 (48%)	24 (53%)	19 (42%)	0.297
In-hospital mortality (n, %)	22 (24%)	17 (38%)	5 (11%)	0.003

**Elective surgery**	Overall n=56	Cirrhosis n=28	Transplantation n=28	p

Length of hospital stay (days, mean±SD)	15.4 ± 10.7	17.9 ±10.4	13.3±10.7	0.079
Morbidity (n, %)	21 (38%)	12 (43%)	9 (32%)	0.41
In-hospital mortality (n, %)	11 (21%)	7 (25%)	4 (14%)	0.32

**Emergency surgery**	Overall n=34	Cirrhosis n=17	Transplantation n=17	p

Length of hospital stay (days, mean±SD)	22.0±18.8	28.3±10.4	19.3±21.2	0.047
Morbidity (n, %)	22 (65%)	12 (71%)	10 (59%)	0.488
In-hospital mortality (n, %)	11 (32%)	10 (59%)	1 (6%)	0.001

**MELD <10**	Overall n=34	Cirrhosis n=17	Transplantation n=17	p

Length of hospital stay (days, mean±SD)/span>	15.8±10.8	16.8±9	14.9±12.6	0.27
Morbidity (n, %)	13 (38%)	6 (35%)	7 (41%)/span>	0.63
In-hospital mortality (n, %	7 (21%)	4 (24%)	3 (18%)	0.75

**MELD 10-14**	Overall n=34	Cirrhosis n=17	Transplantation n=17	p

Length of hospital stay (days, mean±SD)	18±12.1	19.5±10.4	16.7±13.6	0.254
Morbidity (n, %)	14 (41%)	7 (41%)	7 (41%)	1
In-hospital mortality (n, %)	6 (18%)	4 (24%)	2 (12%)	0.38

**MELD >14**	Overall n=22	Cirrhosis n=11	Transplantation n=11	P

Length of hospital stay (days, mean±SD)	23.6±20.6	34±5.7	23±19.7	0.02
Morbidity (n, %)	15 (68%)	9 (82%)	6 (55%)	0.18
In-hospital mortality (n, %)	8 (36%)	7 (64%)	1 (9%)	0.07

Outcomes for each indication and procedure are listed in supplementary Table 1. Stratification analysis and multivariate regression did not show significance.

## Discussion

Surgery in cirrhotic patients carries significant risks, necessitating careful consideration [2, 3]. While liver transplantation offers definitive treatment for end-stage liver disease, perioperative risks remain elevated post-transplantation compared to non-transplanted populations [8–12]. This complexity makes finding the optimal timing for necessary surgery a challenging decision, requiring individualized assessment. The Model for End-Stage Liver Disease (MELD) score appears to be a valuable tool for perioperative risk assessment in this context [6, 7].

The overall elevated risks observed in our cohort for both cirrhotic and transplant recipients reflect the selection of high-risk patients, as indicated by the high mean Charlson Comorbidity Index (CCI) of 6.3. For context, a recent study on the prognostic value of the CCI in colorectal surgery found that a CCI ≥ 3 was associated with a significantly increased risk of in-hospital mortality (OR 16.83 [95% CI 2.23–126.88], *p* = 0.006) [13]. Our findings align with a current meta-analysis of 16 studies for colorectal surgery in cirrhosis patients, which reported mortality rates up to 37% and morbidity rates ranging from 29 to 75%, consistent with our observed rates of 38% and 53%, respectively [14].

As previously mentioned, the increased perioperative risk for cirrhosis patients is well-described in numerous studies [2, 3, 5, 6], yet data on transplant recipients remain scarce. A general increase in perioperative risk is described for kidney transplant recipients [8, 9]for colectomy due to diverticulitis [10, 12] and for pancreatic surgery in transplant recipients [15, 16]. Pathophysiologically, immunosuppression in transplant patients can increase surgical site infections due to impaired wound healing, potentially obscure clinical signs of complications [17] and contribute to an increased number of cardiovascular events [18]. While a degree of immune dysfunction is also described in cirrhosis patients [19]surgery is often unavoidable, particularly given that the prognosis for colorectal cancer is poorer in transplant recipients [20]. Although immunosuppressants like mycophenolic acid are often switched to less harmful agents in elective cases, this is not feasible in emergencies. Interestingly, our study indicates that emergency surgery in cirrhotic patients carries a higher risk than in transplant patients without immunosuppression adjustment due to emergency setting. It is worth noting that while elective surgery showed a trend towards worse outcomes in cirrhotic patients, this did not reach statistical significance.

The heterogeneity of indications and procedures represents a major limitation of our study, which must be carefully considered when interpreting our results. Malignancy was the most common indication for surgery in cirrhotic patients (42%) compared to transplant patients (20%). This difference is notable given that transplant patients are typically screened for carcinoma before transplantation but face an increased risk of malignant neoplasia due to long-term immunosuppression. Conversely, transplant patients more frequently experienced obstruction, possibly due to adhesions from prior liver transplantation. Furthermore, procedures varied between both groups. However, the relatively small sample size limited our ability to additionally match for specific surgical procedures, necessitating a degree of generalization. Tests for confounding did not show relevant dependencies for indications and procedures, which might be caused by small sample size, a residual confounding from unmatched indications and procedures remains. While our case-matched analysis enhanced comparability, a propensity score matching approach, though capable of including more covariates, would have further reduced statistical power. The robustness of findings, particularly within the MELD score subgroups, is thus limited by the small sample size and inherent heterogeneity.

The association between MELD score and perioperative risk in our cohort is consistent with previous research. In our study, each unit increase in MELD score was associated with a 10% relative increase in mortality in cirrhotic patients. A larger analysis of all cirrhotic patients undergoing general surgery at our center previously showed an even higher 20% increase in mortality per MELD point [3]. This dependency has been similarly described by Northup and coworkers [7]. Crucially, cirrhotic patients with MELD scores below 14 had comparable outcomes to transplant recipients.

Even considering these limitations, our findings provide valuable insights that can support individualized decision-making in this challenging patient population. Our study addresses a clinically relevant, yet unanswered, question regarding whether postponing necessary surgery until after liver transplantation is safer for cirrhotic patients.

## Conclusions

Our findings highlight the increased perioperative risk in both cirrhotic and liver transplant recipients undergoing small bowel and colorectal surgery. Particularly in emergency settings and for cirrhotic patients with high MELD scores, there is a significantly increased risk compared to transplant recipients. The MELD score is a valuable tool for risk stratification and can help identify patients for whom delaying surgery until after transplantation may be a safer strategy. Furthermore, elective surgery in cirrhotic patients with low MELD scores (< 14) appears to pose a comparable risk to that in transplant recipients.

## Supplementary Information

Below is the link to the electronic supplementary material.

Supplementary Material 1 (DOCX 16.3 KB)

## Data Availability

The datasets analysed during the current study are not publicly available due to patient privacy limitations, but are available from the corresponding author on reasonable request.

## Abbreviations

MELD: Model for Endstage Liver Disease
TX: Transplant patients
CCI: Charlson Comorbidity Index
CI: Confidence Interval
LOS: Length of hospital stay
OR: Odds Ratio
ns: not significant
