# Supplementary material for: Is it safer to perform surgery before or after liver transplantation? A case-match study for colorectal and small-bowel surgery
Source: Langenbecks Arch Surg. 2025 Sep 27;410(1):280. doi: 10.1007/s00423-025-03858-7 (PMC12476395; doi:10.1007/s00423-025-03858-7)
Supplement: Supplementary file 1 — Supplementary Material 1 (DOCX 16.3 KB) [file 423_2025_3858_MOESM1_ESM.docx]

**Supplementary Table 1: Outcome sorted by Indications and procedures**

|  | Overall  Mortality | Morbidity | Cirrhosis  Mortality | Morbidity | Transplantation  Mortality | Morbidity |
| --- | --- | --- | --- | --- | --- | --- |
| Malignancy | 7 (25%) | 13 (46.4%) | 6 (31.6%) | 11 (57.9%) | 1 (11.1%) | 2 (22.2%) |
| Perforation | 6 (28.6%) | 13 (61.9%) | 5 (38.5%) | 7 (53.8%) | 1 (12.5%) | 6 (75%) |
| Obstruction | 3 (17.6%) | 6 (35.3%) | 1 (20%) | 1 (20%) | 2 (16.7%) | 5 (41.7%) |
| Bleeding | 0 | 0 | 0 | 0 | 0 | 0 |
| Inflammation | 3 (23.1%) | 6 (35.3%) | 3 (60%) | 3 (60%) | 0 | 3 (37.5%) |
| Other | 3 (37.5%) | 5 (62.5%) | 2 (100%) | 2 (100%) | 1 (16.7%) | 3 (50%) |
|  |  |  |  |  |  |  |
| Small bowel resection | 7 (20.6%) | 18 (52.9%) | 4 (30.8%) | 7 (53.8%) | 3 (14.3%) | 11 (52.4%) |
| Hemicolectomy right | 6 (33.3%) | 9 (50%) | 5 (38.5%) | 8 (61.5%) | 1 (20%) | 1 (20%) |
| Sigma resection | 3 (21.4%) | 5 (35.7%) | 3 (33.3%) | 4 (44.4%) | 0 | 1 (20%) |
| Total colectomy | 0 | 2 (22.2%) | 0 | 0 | 0 | 2 (33.3%) |
| Other | 6 (40%) | 9 (60%) | 5 (71.4%) | 5 (71.4%) | 1 (12.5%) | 4 (50%) |
